# Supplementary figures and images for: Microvascular Metrics on Diabetic Retinopathy Severity: Analysis of Diabetic Eye Images from Real-World Data
Source: Biomedicines. 2024 Dec 2;12(12):2753. doi: 10.3390/biomedicines12122753 (PMC11673885; doi:10.3390/biomedicines12122753)

Supporting Material

Figure SI.1:

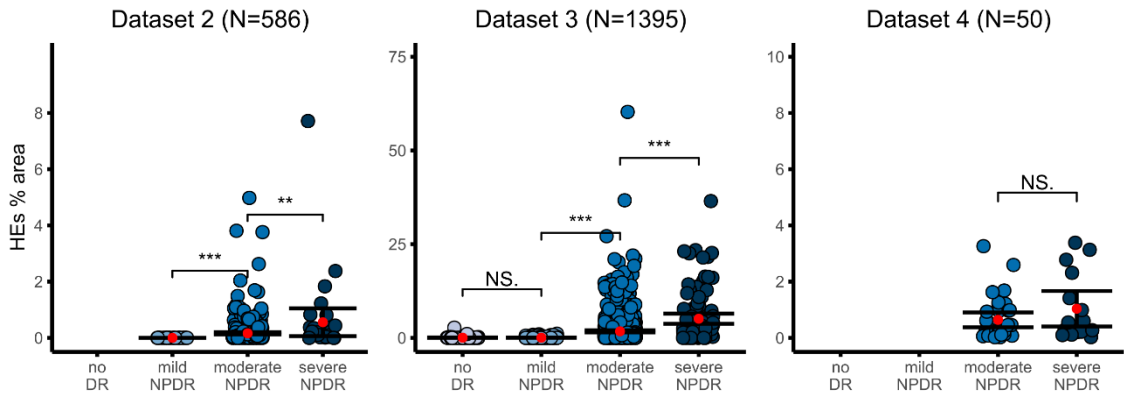

Supplement: Supplementary file 1 [file biomedicines-12-02753-s001.zip › biomedicines-3304780-supplementary.pdf]
